# Analysis of the Healthcare MERS-CoV Outbreak in King Abdulaziz Medical Center, Riyadh, Saudi Arabia, June–August 2015 Using a SEIR Ward Transmission Model

**DOI:** 10.3390/ijerph17082936

**Published:** 2020-04-23

**Authors:** Tamer Oraby, Michael G. Tyshenko, Hanan H. Balkhy, Yasar Tasnif, Adriana Quiroz-Gaspar, Zeinab Mohamed, Ayesha Araya, Susie Elsaadany, Eman Al-Mazroa, Mohammed A. Alhelail, Yaseen M. Arabi, Mustafa Al-Zoughool

**Affiliations:** 1 School of Mathematical and Statistical Sciences, University of Texas Rio Grande Valley, Edinburg, TX 78539, USA; adrianaquirozg@hotmail.com (A.Q.-G.); zeinab.mohamed@huskers.unl.edu (Z.M.); 2 McLaughlin Centre for Population Health Risk Assessment, Faculty of Medicine, University of Ottawa, Ottawa, ON K1N 6N5, Canada; mtyshenk@gmail.com; 3 World Health Organization, 01211 Geneva, Switzerland; balkhyh@hotmail.com; 4 Solid Organ Transplant, University of Texas Southwestern Medical Center, Dallas, TX 75390, USA; yasar.tasnif@utsouthwestern.edu; 5 Valley Baptist Medical Center, Brownsville, TX 78520, USA; aaaraya@utexas.edu; 6 Department of Pathology and Laboratory Medicine, Faculty of Medicine, University of Ottawa, Ottawa, ON K1H 8M5, Canada; susie_elsaadany@hotmail.com; 7 Infection Prevention and Control Department, King Abdulaziz Medical City, Riyadh 14611, Saudi Arabia; MazroaE@ngha.med.sa; 8 King Abdullah International Medical Research Center (KAIMRC), Riyadh 11481, Saudi Arabia; 9 College of Medicine, King Saud bin Abdulaziz, University for Health Sciences, Riyadh 14611, Saudi Arabia; alhelail@yahoo.com (M.A.A.); yaseenarabi@yahoo.com (Y.M.A.); 10 Emergency Department, King Abdulaziz Medical City, Riyadh 14611, Saudi Arabia; 11 Intensive Care Department, King Abdulaziz Medical City, Riyadh 14611, Saudi Arabia; 12 Department of Environmental and Occupational Health, Faculty of Public Health, Kuwait University, Safat 13110, Kuwait; Mustafa.alzoughool@hsc.edu.kw

**Keywords:** healthcare MERS-CoV outbreak, SEIR ward transmission model, basic reproduction number

## Abstract

Middle East respiratory syndrome coronavirus (MERS-CoV) is an emerging zoonotic coronavirus that has a tendency to cause significant healthcare outbreaks among patients with serious comorbidities. We analyzed hospital data from the MERS-CoV outbreak in King Abdulaziz Medical Center, Riyadh, Saudi Arabia, June–August 2015 using the susceptible-exposed-infectious-recovered (SEIR) ward transmission model. The SEIR compartmental model considers several areas within the hospital where transmission occurred. We use a system of ordinary differential equations that incorporates the following units: emergency department (ED), out-patient clinic, intensive care unit, and hospital wards, where each area has its own carrying capacity and distinguishes the transmission by three individuals in the hospital: patients, health care workers (HCW), or mobile health care workers. The emergency department, as parameterized has a large influence over the epidemic size for both patients and health care workers. Trend of the basic reproduction number (R_0_), which reached a maximum of 1.39 at the peak of the epidemic and declined to 0.92 towards the end, shows that until added hospital controls are introduced, the outbreak would continue with sustained transmission between wards. Transmission rates where highest in the ED, and mobile HCWs were responsible for large part of the outbreak.

## 1. Introduction

Middle East respiratory syndrome coronavirus (MERS-CoV) was first reported in Saudi Arabia in 2012 and has led to significant healthcare outbreaks. It has since reported in several other countries as a result of travel-related transmission [1] and in one incident has led to a large outbreak with significant impact in Korea [2]. The European Centre for Disease Prevention and Control (ECDC) classified the following countries in the Middle East as “at-risk” for MERS-CoV: Bahrain, Jordan, Iran, Kuwait, Iraq, Lebanon, Yemen, United Arab Emirates, Oman, Qatar, Saudi Arabia, and Palestine [3]. According to the World Health Organization (WHO) update in February 2019, since MERS-CoV was first reported there have been 2279 lab-confirmed cases worldwide, and 806 associated deaths. The majority (83.4%) of these cases have occurred in the Kingdom of Saudi Arabia [4]. 

MERS-CoV infections can be asymptomatic/mild for some patients but fatal to those with respiratory and/or comorbid conditions. The incubation period ranges from 2–13 days with a median of 5 days. MERS-CoV presents as a combination of non-specific symptoms such as cough, fever (≥38 °C), dyspnea, diarrhea, and vomiting [5]. To distinguish a probable or confirmed case, a combination of symptoms and lab-confirmed data is necessary. The Centers for Disease Control and Prevention (CDC) define two categories of laboratory tests used to detect MERS-CoV: (1) molecular tests, which look for active infection; and (2) serology tests, which look for previous infection [6]. Most, if not all hospitals, use real-time reverse-transcription polymerase chain reaction (rRT-PCR) assays to define a confirmed case following CDC’s standard. The CDC recommends conducting multiple tests on each patient. A single negative rRT-PCR would classify a patient as under investigation, or as a possible case. A second negative would classify the patient as MERS negative [6]. The Saudi Ministry of Health has adopted strict case definitions for the hospitals and created a wave of auditing and inspections to the hospitals to ensure compliance with infection control practices to prevent further healthcare outbreaks. 

The objective of this work is to mathematically model MERS-CoV transmission between several hospital wards using parameters and data from the King Abdulaziz Medical City-Riyadh (KAMC-R), Saudi Arabia. Modelling will provide insights into healthcare transmission and parameters that may be most influential in reducing future hospital outbreaks. Data from the 102-day long healthcare MERS-CoV outbreak that occurred in King Abdulaziz Medical City-Riyadh (KAMC-R), Saudi Arabia from June to August 2015 was used to calibrate the mathematical model. 

## 2. Materials and Methods 

### 2.1. Summary of the June 2015 Healthcare MERS-CoV Outbreak

Following the identification of the virus in 2012 and the following multiple outbreaks in many of the hospitals of the Kingdom, KAMC-R developed an infectious disease epidemic plan (IDEP) to manage the potential influx of cases. Prior to 2015, only sporadic cases were managed and there were no outbreaks in the hospital, other than a cluster in 2013 [7]. In August of 2015, the hospital experienced the second largest reported outbreak of MERS-COV. The IDEP plan, which was released early 2014, stated a three-phase escalation plan to be implemented during a hospital outbreak of MERS-CoV. 

In Riyadh the hospital followed the infectious disease epidemic plan (IDEP), established by the hospital outbreak committee based on CDC and World Health Organization guidelines. Phase I activates when there are up to five suspected/confirmed cases; Phase II activates with 6–30 suspected/confirmed cases; and Phase III activates if there are more than 30 suspected or confirmed cases. Under IDEP Phase I MERS-CoV cases requiring intubation were placed into negative pressure isolation rooms and were cohorted in one intensive-care unit (ICU). All hospital services continued without interruptions except for MERS patients’ precautions in the designated ICU. IDEP Phase II included several actions including (1) conversion of an entire ward into a negative pressure, isolation unit for the treatment of suspected and confirmed non-ICU cases; (2) restricting visitors entering the hospital; (3) reducing the number of elective surgeries; (4) implementing contact tracing to unprotected health care workers (HCWs) and staff; and (5) ensuring mask and PPE fit testing for HCWs in high risk areas. IDEP Phase III implemented additional movement restrictions with cancellation of all elective cardiac surgeries and limiting outpatient clinic visits to urgent visits only [8].

The plan accounted for a conversion of resources in the event of increased need for beds, staff, and equipment. IDEP also considers the safety of the hospital staff as a priority, requiring adherence to immunizations, N95 mask fit testing, and control steps in the event of infection [9]. 

Five cases of acute respiratory failure were admitted to the ICU and subsequently diagnosed with MERS-CoV. IDEP was activated on August 2, initiating strict infection control measures, which included droplet and airborne isolation for suspected and confirmed cases. With the increasing number of identified cases and contacts to be screened, Phase III of IDEP was activated on August 18, starting by closing the emergency department (ED) and creating a diversion plan for the patients with the assistance of the Saudi Ministry of Health, cancelling elective surgeries and closing the outpatient clinic and ED.

Family visits were restricted from an open-policy to 2 h limits, and visitors were not allowed into the patients’ rooms. MERS-CoV confirmed cases were identified separately from MERS-CoV suspected cases and an entry log into the unit was developed, measuring HCW temperature on a daily basis, and a buddy system for the donning and doffing of PPEs for all those entering the designated ward for MERS-CoV confirmed cases. Staff contact was also reduced to include only those necessary. Nurse to patient ratio was increased from 1:1.2 to 1:0.8 and floating nurses between wards were prevented for the duration of the outbreak as much as possible. ICU capacity of negative pressure rooms was tripled, and infection control protocols, along with personal protective equipment (PPE) use, were adaptive and increasingly stringent as the outbreak proceeded [8,9]. 

King Abdulaziz Medical Center, Riyadh, Saudi Arabia used rRT-PCR assays to confirm cases and considers three negative rRT-PCR results to be negative for MERS. There were 130 lab-confirmed cases, and a 63.4% mortality rate. All MERS-CoV infected healthcare workers (HCW) survived. Researchers concluded the necessity of preparation and awareness in all hospital systems [9].

During the MERS-CoV outbreak the movement of people (patients, healthcare workers, and staff) between hospital units appears to be the main contributing factor to the outbreak severity and duration. For the analysis, we used the available transmission data from the outbreak provided by the hospital [9].

### 2.2. Model Description

A SEIR-ward model has been used by researchers for infectious disease modeling spread of coronavirus [10,11,12]. The model uses the key epidemiological features from the MERS-CoV outbreak in KAMC-R that occurred in summer 2015 focusing on human-to-human and environment-to-human transmission in the hospital setting [13]. The nature of the model is important to consider as many large hospital infectious disease outbreaks, like the Saudi Arabia’s MERS-CoV outbreak, often show low disease incidence due to the limited number of individuals (patients and health care workers) in the hospital during outbreak events.

**Figure 1:**
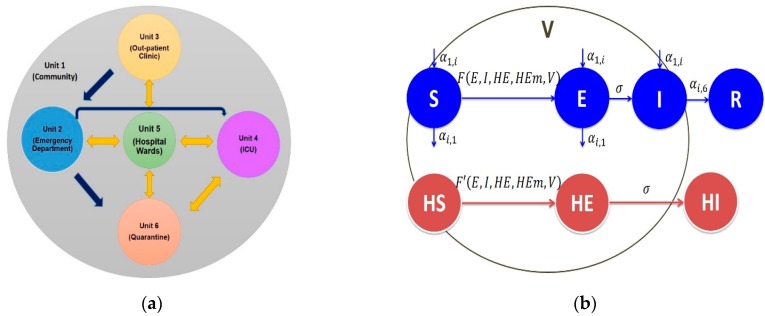
Schematic illustrations of the model. (**a**) Hospital ward model showing the possible movements between wards in the health care facility. (**b**) Hospital ward model movement of people between wards within the health care facility. The parameters $\alpha_{i,j}$ are the transition rates from unit i to unit j. The functions F and $F^{\prime }$ are the forces of infection imposed on susceptible patients and health care workers (HCWs), respectively. The mobile health care workers follow the same transitions between the disease compartments as other health care workers. The rate of isolation $\alpha_{i,6}=:\alpha_{6}$ is assumed to be the same for any unit i. Finally, σ is the rate at which exposed people become symptomatic.

We developed a SEIR-ward model comprised of a system of differential equations for the healthcare MERS-CoV outbreak by dividing the hospital into four units/wards: emergency department (ED), out-patient clinic (OC), intensive care unit (ICU), and general hospital wards (H). Additionally, the model also accounts for a Quarantine-Isolation (Q) that is not necessarily a physical space; it is the status of the disease when infected individuals are not transmitting the disease anymore since some individuals in the model might be quarantined in a different hospital (Figure 1a). We considered these units/wards as batches where transmission of MERS-CoV between individuals [14]. 

The individuals in the model are the patients (P), health care workers (HCW), and mobile health care workers (HCWm). Under the conditions of our SEIR-ward model in a closed population, mobile health care workers travel between wards (units) and are considered to contribute to the nosocomial transmission and the spread between wards. The individuals are divided into eight compartments based on their disease status: S is the proportion of susceptible patients, E is the proportion of exposed patients who are asymptomatic but can transmit the disease to a lesser degree, and I is the proportion of infected (symptomatic) patients. For health care workers, HS is the proportion of susceptible HCW, HE is the proportion of exposed HCW who are asymptomatic but can transmit the disease to a lesser degree, and HI is the proportion of infected HCW. Similar notations are used for mobile HCWs (with an extra subscript m). The quantity V is the viral load in the environment, and Q is quarantined individuals; with all those quantities indexed by their respective unit (Figure 1b). The dynamics due to direct contact between people and indirect environmental transmission are in the Supplementary Materials.

### 2.3. Calculating $R_{0}$

The model has a disease-free equilibrium (DFE) where the infection vanishes. The basic reproduction number $R_{0}$ is defined to be the average number of secondary cases generated by a primary case in an entirely susceptible population [15]. It determines if the disease will have a chance to be epidemic when $R_{0}>1$ or if the disease will cease to spread (DFE is asymptotically stable) if $R_{0}<1$. In the underlying paper, the calculation of $R_{0}$ was done using the next generation matrix $K=F\;V^{-1}$ [16]. We formulated the matrix ℱ that considers the inputs due to infections and the matrix V that considers the outputs or movements of individuals (see Supplementary Materials). Then the two matrices F and V are calculated as the Jacobian of ℱ and V evaluated at the DFE, respectively. The basic reproduction number is the spectral radius of the matrix $K=F\;V^{-1}$; that is, $R_{0}=\rho \left(K\right)$. Due to the large dimension of the matrix K, calculating the spectral radius was performed numerically using Matlab.

### 2.4. Model Calibration by Data Fitting

We assume time dependent transmission rates in the form of a step function, to reflect the response of the epidemic to control measures. That is, patient-to-patient transmission rates are assumed to be given by

(1) $$\beta_{i}\left(t\right)=\{\begin{array}{c} \beta_{i,1},\;\;t\leq t_{i}\\ \beta_{i,2},\;\;t>t_{i} \end{array}$$

for i=2, 3, 4, 5 when 0≤t≤102; HCW’s transmission rates are assumed to be given by

(2) $$\beta_{i,H}\left(t\right)=\{\begin{array}{c} \beta_{i,H,1},\;\;t\leq t_{i,H}\\ \beta_{i,H,2},\;\;t>t_{i,H} \end{array}$$

for i=2, 3, 4, 5 when 0≤t≤102; and mobile HCW’s transmission rates are assumed to be given by

(3) $$\beta_{m}\left(t\right)=\{\begin{array}{c} \beta_{m,1},\;\;t\leq t_{m}\\ \beta_{m,2},\;\;t>t_{m} \end{array}$$

when 0≤t≤102.

To avoid un-identifiability in the estimates of the environmental transmission rates, the virus shedding rate ρ is assumed to be one and the rates $\beta^{\prime }$, which we still call environmental transmission rates for brevity, are the transmission rates multiplied by the virus shedding rate into the environment. Getting infected through the environment is, in essence, a consequence of a delayed transmission from other exposed and infected patients, HCWs, and mobile HCWs. While the model is introduced in its general form, we identified 20 parameters that can be estimated from the epidemic data while the rest are found in the literature or estimated from an independent data provided by KAMC if they are not related to the epidemic, see Tables S1–S3 in the Supplementary Materials. The data size over the 102 days in the four units is relatively larger than the number of parameters in the different units.

The number of beds and HCWs working in the hospital, transitions of patients between the wards, as well as the number of infected individuals and times of their diagnosis were used. The parameters’ values were then estimated using the method of ordinary least squares (OLS) with the objective function given by the sum of squares of difference between the actual proportion of infected patients and the numerical solution of the system of ordinary differential equations, (ODEs). The system of ODEs was solved using Runge–Kutta methods of hybrid order 4 and 5 in MatLab. The OLS was coded in MatLab and the initial values of the optimization algorithm were found using the smallest sums of squares of errors for different random samples from the parameters space performed using the method of Latin-hypercube. The optimization was run from different initial values to make sure of a global optimal solution was reached. The uncertainty in the parameter estimates were estimated using the sensitivity matrices of the curves to the parameter values [17].

### 2.5. Sensitivity Analysis

We carried out local and global sensitivity analyses for $R_{0}$ and the sizes of epidemics. The goal was to determine the most influential factors that control the healthcare-acquired MERS-CoV. 

To locally measure how much the size of epidemic of patients (SIP), HCWs (SIH), mobile HCWs (SIM), and the basic reproduction number $R_{0}$ are sensitive to each parameter at the estimated values we used the local sensitivity measure given by

(4) $$GS_{\theta_{k}}^{K}={\left[\frac{d\log\left(K\left(\Theta \right)\right)}{d\log\left(\theta_{k}\right)}\right]}^{-1}\mathrm{for}\;k=1,2,\;\ldots ,\;p$$

with K being SIP, SIH, SIM, or $R_{0}$; and $\Theta =\left(\theta_{1},\theta_{2},\;\ldots ,\;\theta_{p}\right)$ is the parameter vector. A change of $GS_{\theta_{k}}^{K}100\%$ in the value of the parameter $\theta_{k}$ results in an increase of 1% in K. A global sensitivity analysis was done using Latin-hypercube sampling and the partial rank correlation coefficient (PRCC) between the parameters and K. 

## 3. Results

The model resulted in a good fit to the outbreak under movement of patients in most of the units (see Figures S1–S3 and Tables S1–S3 in Supplementary Materials). According to the model, the potency of the healthcare outbreak in KMAC-R had evolved over its course in the summer 2015 due to the changes in the transmission rates (estimated) ensued by the control plan IDEP. It seems, however, that transmission rates have increased in two wards, ED and hospital, after 26 and 91 days, respectively, after the admission of the index case (see Figure S3a). 

**Figure 2:**
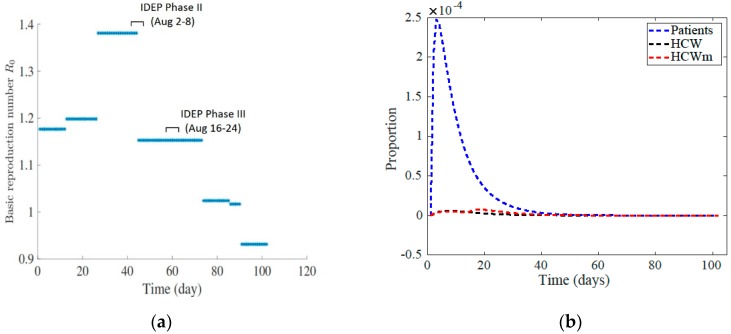
The strength of the outbreak. (**a**) The basic reproduction number $R_{0}$ at different times during the King Abdulaziz Medical City (KAMC) outbreak. (**b**) Evolution of proportion of infected patients, HCW, and HCWm for transmission rates at the levels of the last 10 days of the outbreak. The infection disease epidemic plan phase II (infectious disease epidemic plan (IDEP) II activated August 2–8) is on the time period days 43–49 on the time scale and phase III (IDEP III activated August 16–22) is on the time period days 57–63 on the time scale.

According to the model, the basic reproduction number increased until almost half of the epidemic period and then dropped back two times and decreased below the threshold of one upon the escalation of the infectious disease plan in the hospital (Figure 2a). The IDEP phase changes were activated by the confirmed number of MERS cases not by the R_0_ value. The hospital administration would not have known what the R_0_ value was only that case numbers had increased activating Phases II and III. 

If the outbreak had started at the ending levels of transmission rates, when the basic reproduction number was about 0.93, the epidemics would have been much smaller in size and shorter in duration (Figure 2b).

**Figure 3:**
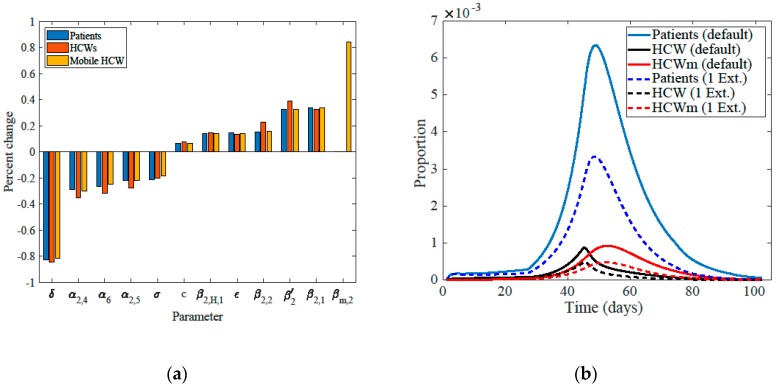
Sensitivity to the different parameters and measures. (**a**) Size of epidemics of patients, HCWs, and mobile HCWs’ sensitivity to the most influential parameters. The parameters from left to right: natural removal rate from environment (δ), transition from the emergency department (ED) to intensive care unit (ICU) ($\alpha_{2,4}$), rate of isolation ($\alpha_{6}$), transition from the ED to the hospital wards ($\alpha_{2,5}$), rate of becoming symptomatic (σ), relative reduction in transmission rates for exposed and asymptomatic (c), transmission rate of HCWs in the ED up to 45 days ($\beta_{2,H,1}$), the parameter ϵ representing relative reduction in transmission from HCWs to patients due to, may be, infection control measures like using masks and gloves, transmission rate of patients in the ED after 26 days ($\beta_{2,2}$), environmental transmission rate in the ED ($\beta_{2}^{\prime }$), transmission rate of patients in the ED up to 26 days ($\beta_{2,1}$), and transmission rate of mobile HCWs after 13 days ($\beta_{m,2}$). The size of the epidemic of patients and HCWs are still sensitive to $\beta_{m,2}$ but to a lesser degree; that is, an increase of 1% in SIP, and SIH requires about 3.6% and 4.4% increase in $\beta_{m,2}$, respectively. (**b**) Evolution of proportion of infected patients, HCW, and mobile health care workers (HCWm) for the default of three times cleaning versus the scenario of one extra-cleaning.

The disease dynamics in the ED, in particular, have a high influence over the whole healthcare outbreak (Figure 3a). The sensitivity bar graph (Figure 3a) shows that fast increase in the sizes of the epidemics would follow from the increase in the transmission rates in the ED. Consistently, they would also increase with the increase in the length of stay in the ED (or equivalently to the decrease in the transition rates out of the ED to the ICU, hospital wards, or isolation). Moreover, they increase with the rates of becoming symptomatic due to the isolation of symptomatic infectious patients, in general, and out of the ED, in particular; noting that asymptomatic patients are still infectious but to a lesser degree. Additionally, increasing the relative reduction of infectiousness from the HCWs to patients may be due to the lack of gloves and gowns, would increase the sizes of the epidemics. Sensitivity analysis shows also that the sizes of the epidemics of patients, HCWs, and mobile HCWs are less sensitive to parameters other than those related to the ED and some disease-specific parameters. In other words, changes less than 1% in the values of the latter parameters are sufficient to increase the sizes of the epidemics by 1%. 

Additionally, the time of natural clearance from the environment was estimated to be 1.3 days and frequent cleaning might have damped that route of infection transmission. If number of, presumably perfect, environmental cleaning is increased by one more than the default of three times a day, then the peak of the epidemics would decrease by about one-half (Figure 3b).

**Figure 4:**
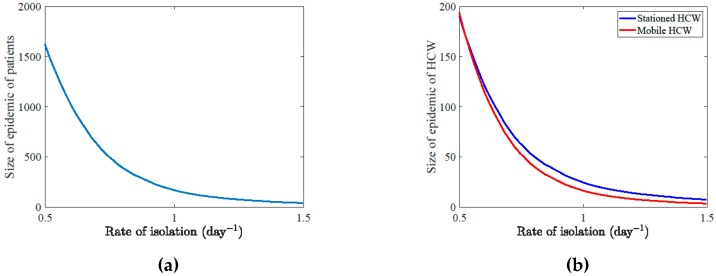

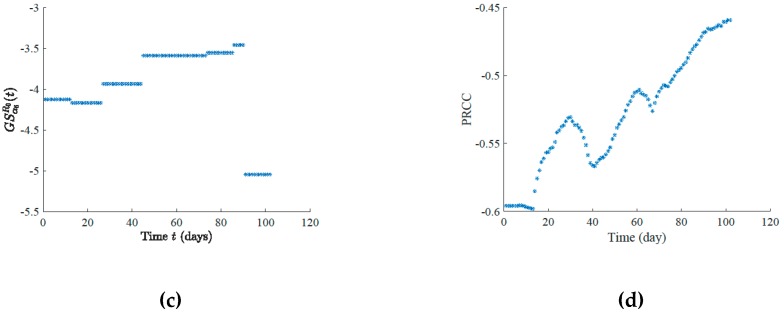
The effect of the rate of isolation on the outbreak. An exponential-like decline in the size of the epidemic if the rate of isolation of suspected cases is within a range of 50% around the estimated value of 0.94 for (**a**) patients and (**b**) both types of HCWs. Local (**c**) and global (**d**) sensitivity of the values of $R_{0}$ over time to the rate of isolation ($\alpha_{6}$). In (**d**) partial rank correlation coefficient (PRCC) of the rate of isolation and $R_{0}$ over time shows a negative significant correlation (*p*-value = 0). That association weakened over time but stayed significant.

The rate of isolation of patients is one of the major factors in suppressing the epidemic (Figure 4a,b). It was estimated that if patients become symptomatic or HCWs are removed from the system before 3.7 days after infection would have greatly influenced the size of the outbreak in the summer 2015 epidemic. The basic reproduction number also had a significant negative association with the rate of isolation during the epidemic (Figure 4c,d). 

**Figure 5:**
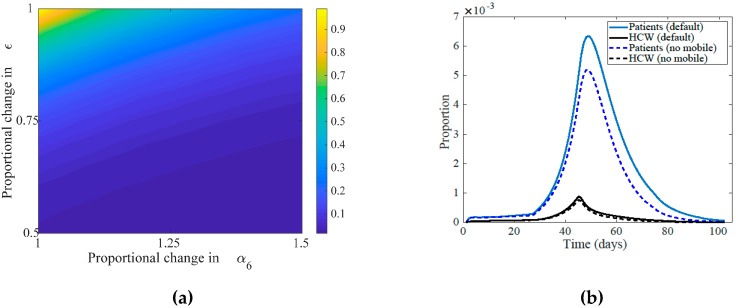
The effect of control measures imposed on patients versus on HCWs. (**a**) Relative total size of epidemics of patients, HCWs, and mobile HCWs’ to the maximum size of epidemic is calculated for increased rate of isolation ($\alpha_{6}$) versus decreased transmission from HCWs to patients due to infection control measures like using masks and gloves (ϵ). It indicates that isolation rate is more efficient than the relative reduction in infectivity due to protective equipment; with smaller relative values attained as $\left(\epsilon ,\alpha_{6}\right)$ gets closer to the (0.5,1.5). (**b**) The sizes of the epidemics of patients and HCWs for the fitted values versus when the HCWm are removed from the system.

Stricter measures taken by HCWs in the hospital, like using added personal protective equipment (gloves, masks, gowns) and more environmental sanitation, may have decreased the number of infected HCWs (Figure 5a). Additionally, it would have been supportive if restricting mobile healthcare workers (HCWm), as they faced relatively higher transmission rates after about 13 days since the beginning of the epidemic or the admission of the index case (Figure 5b).

## 4. Discussion

Potential for human-to-human transmission of MERS-CoV is considered low [18]. Despite this, there have been sustained hospital outbreaks of MERS-CoV due to factors that favor viral transmission within hospital units. Several outbreak case reports have deduced the main reasons for the sustained hospital transmissions with contributing factors of patient comorbidity, patient age, crowded emergency rooms, lack of triage, unawareness of possible MERS-CoV infected patients, and varying levels of adherence to infectious control measures. Thus, the transmission factors of MERS-CoV resulting in healthcare outbreaks is certainly multifactorial. Crowded emergency departments are not uncommon in hospital systems; in these situations, effective acute respiratory infection (ARI) screening is vital to determine patient priority and treatment. The lack of efficient system for bed management resulted in crowding due to boarding within the ED. Other studies conducted in Seoul, Korea and in Saudi Arabia (Jeddah and Riyadh) cite the inadequacy of triage conducted prior to the outbreaks [19,20,21]. 

Further, while MERS-CoV is known and prepared for in the Middle East, as was demonstrated by KAMC-R, air travel may spread the virus to other countries not as familiar with or prepared to deal with MERS-CoV in their hospital systems. This was the case in South Korea’s MERS-CoV outbreak with the delayed recognition and diagnosis of the disease, along with a prolonged exposure to other patients prior to admittance of the index case into isolation [22,23]. Factors such as long-stays in the ED and high mobility of infected patients due to lack of early recognition and diagnosis of the virus contributed to the healthcare acquired infections spread and transmission between gulf cities. Finally, preventative care for the virus remains rudimentary with lack of an efficacious vaccine. The development of preventative care for both humans and dromedary camels as a community vector could potentially decrease or eradicate infections (see Figure S4 in upplementary Materials). 

In contrast to conventional, deterministic modeling approaches, the model described herein is both multi-type and spatial. It incorporates environmental and human behavior variables through time and space that impact disease transmission within multiple hospital wards. The model outputs were validated by comparing actual data to modeled proportions. The underlying SEIR-ward model for KAMC confirms the importance of including additional spatial-specific characteristics to replicate transmission and super spreading events of infectious disease transmission occurring in different hospital wards. That would assist in identifying the most infectious ward. It also quantifies the role of the different individuals in the disease spread. The small number of people in each ward and state might be a problem when using a deterministic model and a stochastic model is warranted; however, the strength of the disease without the control measures was a main factor in our selection.

Results of $R_{0}$ in the current study were close to those reported in previous studies. Previous estimates of an overall $R_{0}$ (which involves both index and secondary cases) for MERS-CoV ranged from 0.42 to 0.92 [24,25,26,27]. In the later study, $R_{0}$ value was 1.5 for index cases resulting from animal-to-human transmission. In another analysis, Chowel et al. [13] showed that index cases have an $R_{i}$ of 0.84 compared to $R_{s}$ of 0.36 for secondary cases, indicating that potential disease transmission is higher for index cases than for secondary cases. An important finding from the study of Chowel et al. (2014) that hospital-based transmission was 4.3–4.6-fold higher than community-based transmission [12,24,25,26,27].

As one index case can cause a healthcare outbreak that takes months to eradicate, prompt control of the outbreak will assist mitigating a spillover to the community and the host population, preventing a reinforcing feedback loop. A thorough investigation of that course should be studied in more detail to help inform policy and provide the appropriate measures needed to mitigate future nosocomial outbreaks.

## 5. Conclusions

According to the model, MERS-CoV transmitted between people at different rates in each department, being highest in the emergency department (ED). It was effective to close the ED department as a required escalation in the infectious disease plan IDEP. In addition, patients’ isolation and reducing contact with HCWs have a great influence on the epidemic. The mobile HCW were responsible for part of the spread of the disease in the hospital, and infection control measures will reduce the size of the outbreak.

## Supplementary Materials

The following are available online at https://www.mdpi.com/1660-4601/17/8/2936/s1, Supplementary Material.pdf.
