# Supplementary material for: Analysis of the Healthcare MERS-CoV Outbreak in King Abdulaziz Medical Center, Riyadh, Saudi Arabia, June–August 2015 Using a SEIR Ward Transmission Model
Source: Int J Environ Res Public Health. 2020 Apr 23;17(8):2936. doi: 10.3390/ijerph17082936 (PMC7215950; doi:10.3390/ijerph17082936)
Supplement: Supplementary file 1 [file ijerph-17-02936-s001.pdf]

Supplementary Material

Analysis of the Healthcare MERS-CoV Outbreak in King Abdulaziz Medical Center, Riyadh, Saudi Arabia, June–August 2015 Using a SEIR Ward Transmission Model

Tamer Oraby [1,*], Michael G. Tyshenko [2], Hanan H. Balkhy [3], Yasar Tasnif [4], Adriana Quiroz-Gaspar [1], Zeinab Mohamed [1], Ayesha Araya [5], Susie Elsaadany [6], Eman Al-Mazroa [7,8], Mohammed A Alhelail [9,10] Yaseen M. Arabi [9,11] and Mustafa Al-Zoughool [12]

[1] School of Mathematical and Statistical Sciences, University of Texas Rio Grande Valley, Edinburg, TX 78539, USA; adrianaquirozg@hotmail.com (A.Q.G.); zeinab.mohamed@huskers.unl.edu (Z.M.)
[2] McLaughlin Centre for Population Health Risk Assessment, Faculty of Medicine, University of Ottawa, Ottawa K1N 6N5, ON, Canada; mtyshenk@gmail.com
[3] World Health Organization, 01211 Geneva, Switzerland; balkhyh@hotmail.com
[4] Solid Organ Transplant, University of Texas Southwestern Medical Center, Dallas, TX 75390, USA; yasar.tasnif@utsouthwestern.edu
[5] Valley Baptist Medical Center, Brownsville, TX 78520, USA; aaaraya@utexas.edu
[6] Department of Pathology and Laboratory Medicine, Faculty of Medicine, University of Ottawa. Ottawa K1H 8M5, ON, Canada; susie_elsaadany@hotmail.com
[7] Infection Prevention and Control Department, King Abdulaziz Medical City, Riyadh 14611, Saudi Arabia; MazroaE@ngha.med.sa
[8] King Abdullah International Medical Research Center (KAIMRC), Riyadh 11481, Saudi Arabia
[9] College of Medicine, King Saud bin Abdulaziz, University for Health Sciences, Riyadh 14611, Saudi Arabia; alhelail@yahoo.com (M.A.A.); yaseenarabi@yahoo.com (Y.M.A.)
[10] Emergency Department, King Abdulaziz Medical City, Riyadh 14611, Saudi Arabia
[11] Intensive Care Department, King Abdulaziz Medical City, Riyadh 14611, Saudi Arabia
[12] Department of Environmental and Occupational Health, Faculty of Public Health, Kuwait University, Safat 13110, State of Kuwait; Mustafa.alzoughool@hsc.edu.kw
* Correspondence: tamer.oraby@utrgv.edu; Tel.: +1-956-665-3536

Table of Contents:

**Content**	**Page**
Hospital Description	3
Some Assumptions about the Model	3
More Findings from the Model	3
Community-Hospital Spillover-Spillback	6
Scenarios SEIR Batch-Ward Model's Equations	8
Tables of Parameters	14
Next Generation Matrix to find R_0	17
References	23

Hospital Description

While speculation has surrounded the origin of the virus for about six years, it has been proposed to be a zoonotic disease hosted by dromedary camels. In the gulf region dromedary camels are the intermediate host for MERS as they carry evidence of sero-positivity for MERS-CoV (1, 2). Evidence shows MERS-CoV can be transmitted to dromedary workers at a high rate (50%) possibly resulting in community cases and healthcare outbreaks (3). King Abdulaziz Medical City-Riyadh (KAMC-R), Saudi Arabia is a 940-bed tertiary care hospital the Eastern side of the 7 million population capital city of Riyadh and is 10 Km from the largest camel market. The hospital consists of the largest ED in the region with 120 beds divided into 5 main areas, receiving at the time around 500 visits a day and an occupancy rate of 120%. There were 36 wards containing single, double and quadruple rooms. Within the hospital there are 56-single negative pressure rooms for isolation of patients: 5 in the ED, 1-2 in the ICU, and some in most wards (4).

Some Assumptions about the Model

For this model, we have interacting agents; patients and health care workers (HCW) who may contract the disease inside the hospital and become infected. We assume HCW are removed (quarantined) once turn symptomatic and then do not contribute to the epidemic, Since HCW, once exposed, are contacted by the IPC department and are educated about the risks and isolation policy to be followed. HCW are expected to understand and follow protocol.

The model is calibrated using the data provided by the hospital. The data fitting is done using nonlinear least squares to estimate the parameters of the model. A global search for optimal values of the parameters of the model was performed on MatLab using "multisearch."

More Findings from the Model

The fitting of the data to the model is attested in Figures S1 and S2. They show the cumulative proportion of infected patients and HCWs at the different times.

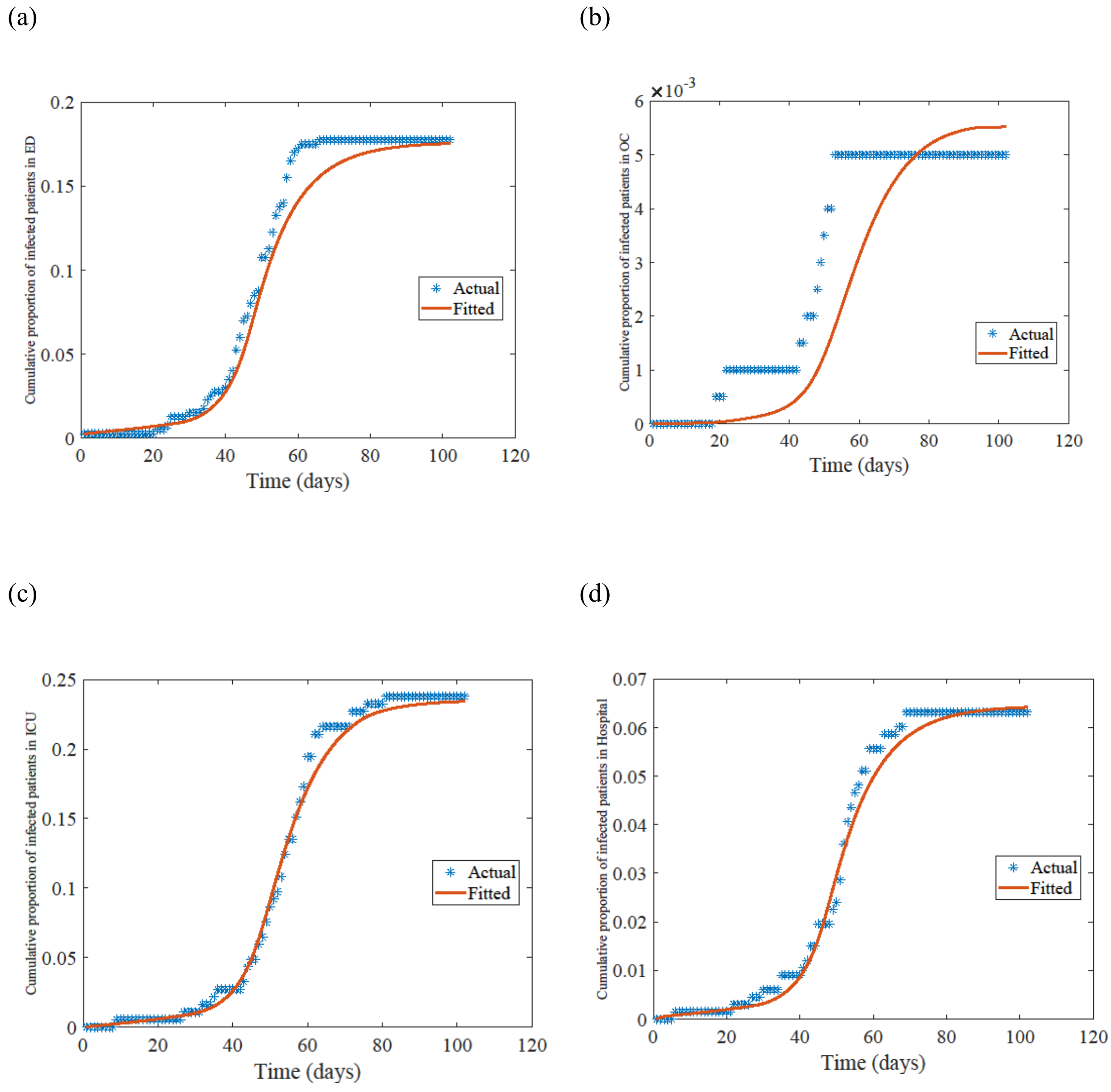

Figure S1. The actual and fitted cumulative proportion of infected patients in (a) emergency department, (b) outpatient clinic, (c) intensive care unit, and (d) hospital wards. The infection disease epidemic plan phase II (IDEP II activated Aug 2-8) is on the time period 43-49 on the time scale and phase III (IDEP III activated Aug 16-22) is on the time period 57-63 on the time scale.

(a)

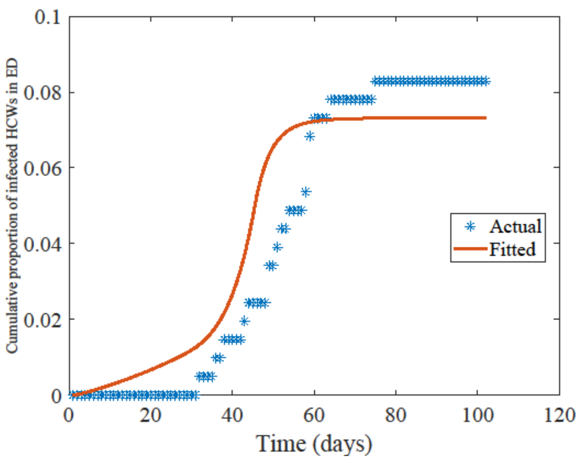

(b)

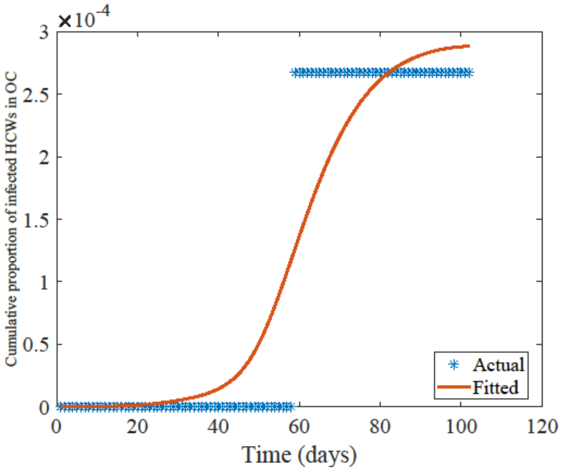

(c)

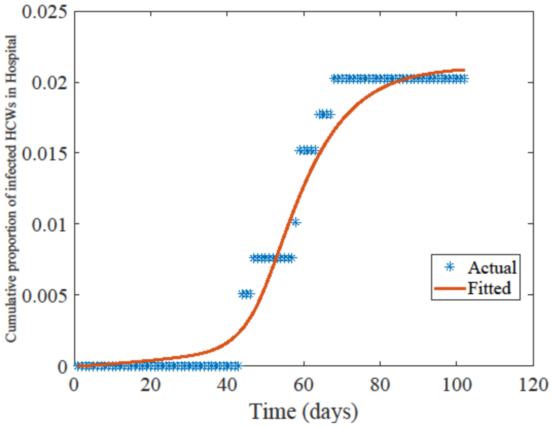

(d)

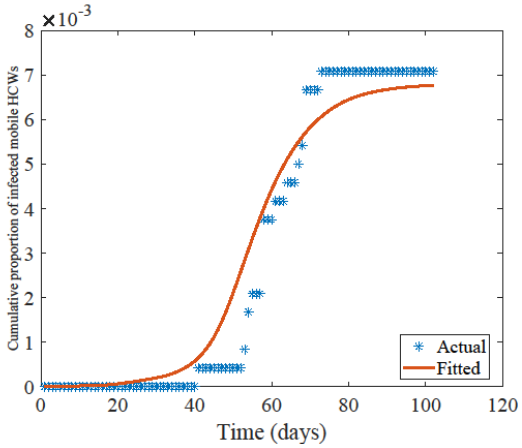

Figure S2. The actual and fitted cumulative proportion of infected health-care workers in (a) emergency department, (b) outpatient clinic, (c) hospital wards, and (d) mobile HCWs. The infection disease epidemic plan phase II (IDEP II activated Aug 2-8) is on the time period 43-49 on the time scale and phase III (IDEP III activated Aug 16-22) is on the time period 57-63 on the time scale.

The epidemic seemingly had different transmission rates in each unit, at different times, and for different agents (Figure S3 a, b, and c). The estimated values of environmental transmission rates to HCWs in the hospital are relatively higher than those of agent-to-agent in the same unit and higher than that of patients.

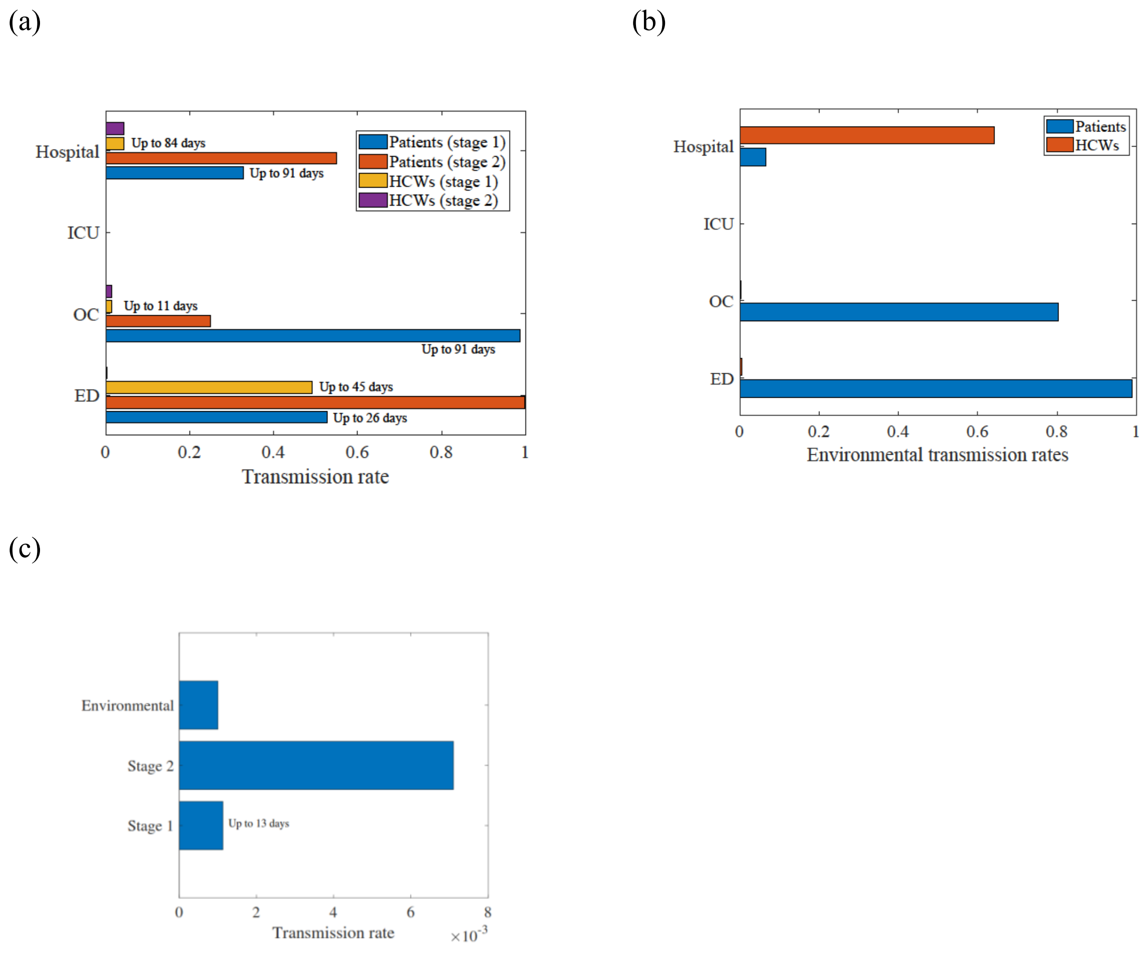

Figure S3. Estimated transmission rates and R_0 over the epidemic period of 102 days. (a) Transmission rates for patients and HCWs; (b) Environmental transmission rates of patients and HCWs in various hospital wards; (c) Environmental transmission rate during stage 1 (to day 13) and stage 2 (day 14 to the end of the KAMC outbreak).

Community-Hospital Spillover-Spillback Scenarios

In this part, under two what-if scenarios, the model is used to investigate the possible occurrence of a spillover community-camel epidemic ensued by a healthcare outbreak and how it can spillback to the hospital. We use healthcare MERS-CoV parameters based on the summer 2015 KAMC outbreak. The rest of the parameters are chosen in a conservative fashion. Since the disease is harder to transmit in the community (5), we assume that transmission rates from an infected patient to visitors and from a community member to another community member are one tenth (scenario: SC 1), and one hundredth (scenario: SC 2) of the smallest patient-to-patient transmission rate inside the hospital throughout the healthcare outbreak. We also assume that there are initially that 564 in 1,000 camels are hosting the virus. Additionally, we assume that infected community members can transfer virus to camels, camels can infect community members, and camel can transfer virus to another camel at the same levels of transmission as in SC1 and SC2 (6). The effect of potent forces of transmission can be extrapolated from the results.

A spillover to the community and a spillback from the community to the hospital can happen (Figure S4 a), even at the-hard-to-transmit scenarios. A spillover to the community might occur causing the nosocomial epidemic to achieve higher levels of transmission (compare Figure S4 b to Figure 5 b in the main text). A spillover to the camels might also occur (Figure S4 c) and in its turn affect the community.

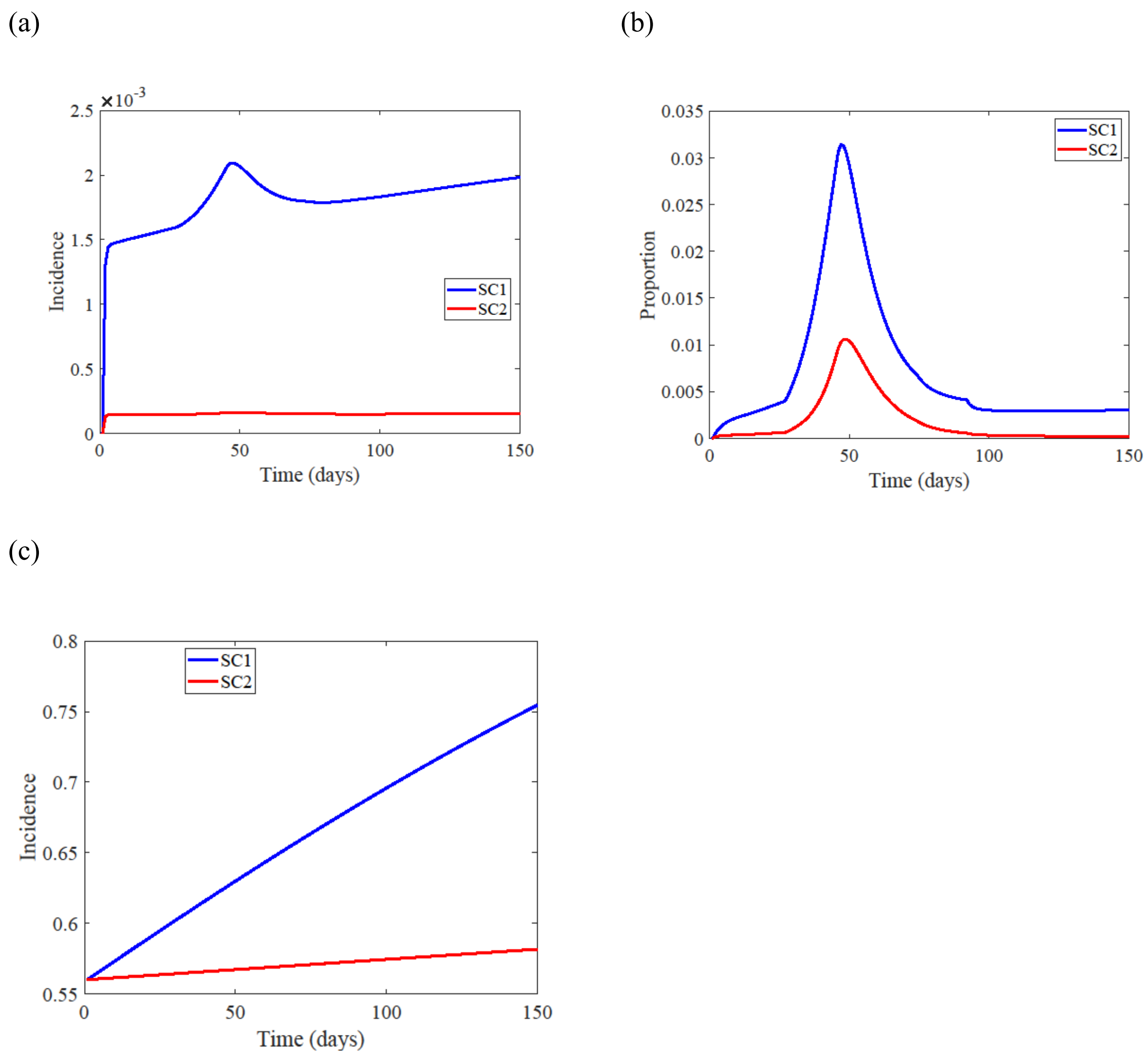

Figure S4. In two what-if scenarios: one-tenth scenario (SC 1) and one-hundredth scenario (SC 2) of minimal transmission rates gives (a) community incidence; (b) proportion of infections in hospital; and (c) incidence of camel hosting.

SEIR Batch-Ward Model's Equations

In this appendix, we will show the seven components of the SEIR-Ward model in which the wards are connected through the movement of patients and mobile HCWs. The first component is the Waiting room and it does not involve HCWs. The last component (Quarantine) is an absorbing part of the process and no disease transmission is allowed as we assume 100% success in controlling the disease in that component. We also assume that it is more of a status than a place or unit the hospital since HCWs can get admitted to a different hospital than their workplace. All wards are assumed to be full at their bed/chair capacity and all work shifts of HCWs are also full.

1) Community/Camel

Infection in community is modeled but not estimated and is only used in a scenario analysis. H is the proportion of hosting camels.

$$\frac{dE_1}{dt} = \beta_c(1-E_1-I_1)H + \beta_0(1-E_1-I_1)(cE_1+I_1) + \beta_1(1-E_1-I_1)(c(E_2 + E_3 + E_4 + E_5) + \epsilon(HE_2 + HE_3 + HE_4 + HE_5) + \xi HE_m + I_2 + I_3 + I_4 + I_5) - (\alpha_{1,2} + \alpha_{1,3} + \alpha_{1,4} + \alpha_{1,5})E_1 - \sigma E_1$$

$$\frac{dI_1}{dt} = \sigma E_1 - (\alpha_{1,2} + \alpha_{1,3} + \alpha_{1,4} + \alpha_{1,5})I_1$$

$$\frac{dH}{dt} = \beta_c(1-H)(cE_1 + I_1) + \beta_{c,c}H(1-H)$$

2) Emergency Department (ED)

$$\frac{dS_2}{dt} = -\beta_2 S_2(c(E_2 + \epsilon HE_2 + \xi HE_m) + I_2) - \beta_2' S_2 V_2 + \alpha_{1,2}S_1 + \alpha_{3,2}S_3 + \alpha_{4,2}S_4 + \alpha_{5,2}S_5 - (\alpha_{2,1} + \alpha_{2,3} + \alpha_{2,4} + \alpha_{2,5})S_2$$

$$\frac{dE_2}{dt} = \beta_2(1-E_2-I_2)(c(E_2 + \epsilon HE_2 + \xi HE_m) + I_2) + \beta_2'(1-E_2-I_2)V_2 + \alpha_{1,2}E_1 + \alpha_{3,2}E_3 + \alpha_{4,2}E_4 + \alpha_{5,2}E_5 - (\alpha_{2,1} + \alpha_{2,3} + \alpha_{2,4} + \alpha_{2,5})E_2 - \sigma E_2$$

$$\frac{dI_2}{dt} = \sigma E_2 + \alpha_{1,2}I_1 + \alpha_{3,2}I_3 + \alpha_{4,2}I_4 + \alpha_{5,2}I_5 - (\alpha_{2,3} + \alpha_{2,4} + \alpha_{2,5} + \alpha_{2,6})I_2$$

$$\frac{dHS_2}{dt} = \sigma HE_2 - \beta_{2,H} HS_2(c(E_2 + \epsilon HE_2 + \xi HE_m) + I_2) - \beta'_{2,H} HS_2 V_2$$

$$\frac{dHE_2}{dt} = \beta_{2,H}(1 - HE_2)(c(E_2 + \epsilon HE_2 + \xi HE_m) + I_2) + \beta'_{2,H}(1 - HE_2)V_2 - \sigma HE_2$$

$$\frac{dHI_2}{dt} = \sigma HE_2$$

$$\frac{dV_2}{dt} = \rho(c(E_2 + \epsilon HE_2 + \xi HE_m) + I_2) - (b_2 + \delta)V_2$$

Such that $0 \leq S_2 + E_2 + I_2 = 1$ and $0 \leq HS_2 + HE_2 = 1$

The unit capacity stability and the complete occupancy assumption entail that:

$$\alpha_{1,2} + \alpha_{3,2} + \alpha_{4,2} + \alpha_{5,2} = \alpha_{2,1} + \alpha_{2,3} + \alpha_{2,4} + \alpha_{2,5}$$

3) Outpatient Clinic (OpC)

$\frac{dS_3}{dt} = -\beta_3 S_3(c(E_3 + \epsilon HE_3 + \xi HE_m) + I_3) - \beta'_3 S_3 V_3 + \alpha_{1,3} S_1 + \alpha_{2,3} S_2 + \alpha_{4,3} S_4 + \alpha_{5,3} S_5 - (\alpha_{3,1} + \alpha_{3,2} + \alpha_{3,4} + \alpha_{3,5}) S_3$

$\frac{dE_3}{dt} = \beta_3(1 - E_3 - I_3)(c(E_3 + \epsilon HE_3 + \xi HE_m) + I_3) + \beta'_3(1 - E_3 - I_3)V_3 + \alpha_{1,3} E_1 + \alpha_{2,3} E_2 + \alpha_{4,3} E_4 + \alpha_{5,3} E_5 - (\alpha_{3,1} + \alpha_{3,2} + \alpha_{3,4} + \alpha_{3,5}) E_3 - \sigma E_3$

$\frac{dI_3}{dt} = \sigma E_3 + \alpha_{1,3} I_1 + \alpha_{2,3} I_2 + \alpha_{4,3} I_4 + \alpha_{5,3} I_5 - (\alpha_{3,2} + \alpha_{3,4} + \alpha_{3,5} + \alpha_{3,6}) I_3$

$\frac{dHS_3}{dt} = \sigma HE_3 - \beta_{3,H} HS_3(c(E_3 + \epsilon HE_3 + \xi HE_m) + I_3) - \beta'_{3,H} HS_3 V_3$

$\frac{dHE_3}{dt} = \beta_{3,H}(1 - HE_3)(c(E_3 + \epsilon HE_3 + \xi HE_m) + I_3) + \beta'_{3,H}(1 - HE_3)V_3 - \sigma HE_3$

$\frac{dHI_3}{dt} = \sigma HE_3$

$\frac{dV_3}{dt} = \rho(c(E_3 + \epsilon HE_3 + \xi HE_m) + I_3) - (b_3 + \delta)V_3$

Such that $0 \leq S_3 + E_3 + I_3 = 1$ and $0 \leq HS_3 + HE_3 = 1$

The unit capacity stability and the complete occupancy assumption entail that:

$$\alpha_{1,3} + \alpha_{2,3} + \alpha_{4,3} + \alpha_{5,3} = \alpha_{3,1} + \alpha_{3,2} + \alpha_{3,4} + \alpha_{3,5}$$

4) Intensive Care Unit (ICU)

$\frac{dS_4}{dt} = -\beta_4 S_4(c(E_4 + \epsilon HE_4 + \xi HE_m) + I_4) - \beta'_4 S_4 V_4 + \alpha_{1,4} S_1 + \alpha_{2,4} S_2 + \alpha_{3,4} S_3 + \alpha_{5,4} S_5 - (\alpha_{4,1} + \alpha_{4,2} + \alpha_{4,3} + \alpha_{4,5}) S_4$

$\frac{dE_4}{dt} = \beta_4(1 - E_4 - I_4)(c(E_4 + \epsilon HE_4 + \xi HE_m) + I_4) + \beta'_4(1 - E_4 - I_4)V_4 + \alpha_{1,4} E_1 + \alpha_{2,4} E_2 + \alpha_{3,4} E_3 + \alpha_{5,4} E_5 - (\alpha_{4,1} + \alpha_{4,2} + \alpha_{4,3} + \alpha_{4,5}) E_4 - \sigma E_4$

$\frac{dI_4}{dt} = \sigma E_4 + \alpha_{1,4} I_1 + \alpha_{2,4} I_2 + \alpha_{3,4} I_3 + \alpha_{5,4} I_5 - (\alpha_{4,2} + \alpha_{4,3} + \alpha_{4,5} + \alpha_{4,6}) I_4$

$\frac{dHS_4}{dt} = \sigma HE_4 - \beta_{4,H} HS_4(c(E_4 + \epsilon HE_4 + \xi HE_m) + I_4) - \beta'_{4,H} HS_4 V_4$

$\frac{dHE_4}{dt} = \beta_{4,H}(1 - HE_4)(c(E_4 + \epsilon HE_4 + \xi HE_m) + I_4) + \beta'_{4,H}(1 - HE_4)V_4 - \sigma HE_4$

$\frac{dHI_4}{dt} = \sigma HE_4$

$\frac{dV_4}{dt} = \rho(c(E_4 + \epsilon HE_4 + \xi HE_m) + I_4) - (b_4 + \delta)V_4$

Such that $0 \leq S_4 + E_4 + I_4 = 1$ and $0 \leq HS_4 + HE_4 = 1$

The unit capacity stability and the complete occupancy assumption entail that:

$$\alpha_{1,4} + \alpha_{2,4} + \alpha_{3,4} + \alpha_{5,4} = \alpha_{4,1} + \alpha_{4,2} + \alpha_{4,3} + \alpha_{4,5}$$

5) Hospital (Wards)

$\frac{dS_5}{dt} = -\beta_5 S_5(c(E_5 + \epsilon HE_5 + \xi HE_m) + I_5) - \beta'_5 S_5 V_5 + \alpha_{1,5} S_1 + \alpha_{2,5} S_2 + \alpha_{3,5} S_3 + \alpha_{4,5} S_4 - (\alpha_{5,1} + \alpha_{5,2} + \alpha_{5,3} + \alpha_{5,4}) S_5$

$\frac{dE_5}{dt} = \beta_5(1 - E_5 - I_5)(c(E_5 + \epsilon HE_5 + \xi HE_m) + I_5) + \beta'_5(1 - E_5 - I_5)V_5 + \alpha_{1,5} E_1 + \alpha_{2,5} E_2 + \alpha_{3,5} E_3 + \alpha_{4,5} E_4 - (\alpha_{5,1} + \alpha_{5,2} + \alpha_{5,3} + \alpha_{5,4}) E_5 - \sigma E_5$

$\frac{dI_5}{dt} = \sigma E_5 + \alpha_{1,5} I_1 + \alpha_{2,5} I_2 + \alpha_{3,5} I_3 + \alpha_{4,5} I_4 - (\alpha_{5,2} + \alpha_{5,3} + \alpha_{5,4} + \alpha_{5,6}) I_5$

$\frac{dHS_5}{dt} = \sigma HE_5 - \beta_{5,H} HS_5(c(E_5 + \epsilon HE_5 + \xi HE_m) + I_5) - \beta'_{5,H} HS_5 V_5$

$\frac{dHE_5}{dt} = \beta_{5,H}(1 - HE_5)(c(E_5 + \epsilon HE_5 + \xi HE_m) + I_5) + \beta'_{5,H}(1 - HE_5)V_5 - \sigma HE_5$

$\frac{dHI_5}{dt} = \sigma HE_5$

$\frac{dV_5}{dt} = \rho(c(E_5 + \epsilon HE_5 + \xi HE_m) + I_5) - (b_5 + \delta)V_5$

Such that $0 \leq S_5 + E_5 + I_5 = 1$ and $0 \leq HS_5 + HE_5 = 1$

The unit capacity stability and the complete occupancy assumption entail that:

$$\alpha_{1,5} + \alpha_{2,5} + \alpha_{3,5} + \alpha_{4,5} = \alpha_{5,1} + \alpha_{5,2} + \alpha_{5,3} + \alpha_{5,4}$$

6) Quarantine (MERS-CoV wards)

$\frac{dQ}{dt} = \alpha_{1,6} I_1 + \alpha_{2,6} I_2 + \alpha_{3,6} I_3 + \alpha_{4,6} I_4 + \alpha_{5,6} I_5$

m) Mobile HCW

$\frac{dHS_m}{dt} = \sigma HE_m - \beta_m HS_m(c(E_2 + E_3 + E_4 + E_5 + \epsilon(HE_2 + HE_3 + HE_4 + HE_5) + \xi HE_m) + I_2 + I_3 + I_4 + I_5) - \beta'_m HS_m(V_2 + V_3 + V_4 + V_5)$

$\frac{dHE_m}{dt} = \beta_m(1 - HE_m)(c(E_2 + E_3 + E_4 + E_5 + \epsilon(HE_2 + HE_3 + HE_4 + HE_5) + \xi HE_m) + I_2 + I_3 + I_4 + I_5) + \beta'_m(1 - HE_m)(V_2 + V_3 + V_4 + V_5) - \sigma HE_m$

$\frac{dHI_m}{dt} = \sigma HE_m$

Such that $0 \leq HS_m + HE_m = 1$

Tables of Parameters

Hospital Parameters

Table S1. King Abdulaziz Medical Center (KAMC), Hospital Parameters, Description and Base Values.

Parameter	Description	Base values	Sources
$\alpha_{i,j}$	Transition rate between the unit i to unit j	Estimated	Independent hospital data
κ_2	Number of beds in unit 2	150	Provided by KAMC
κ_3	Number of patients in unit 3	2000	Provided by KAMC
κ_4	Number of beds in unit 4	185	Provided by KAMC
κ_2'	Number of HCW in unit 2	205	Provided by KAMC
κ_3'	Number of HCW in unit 3	3741	Provided by KAMC
κ_4'	Number of HCW in unit 4	1270	Provided by KAMC
κ_5'	Number of HCW in unit 5	395	Provided by KAMC
κ_m'	Number of mobile HCW	2400	Provided by KAMC
b_2	Cleaning rate for unit 2	3	Provided by KAMC
b_3	Cleaning rate for unit 3	3	Provided by KAMC
b_4	Cleaning rate for unit 4	3	Provided by KAMC
b_5	Cleaning rate for unit 5	3	Provided by KAMC

Disease Parameters

Table S2. Disease Parameters, Description and Base Values.

Parameter	Description	Base values (SD)	Sources
β_2	Human to human transmission rate from unit 2	.53(.26)	Estimated
β_3	Human to human transmission rate from unit 3	.98(2.71)	Estimated
β_4	Human to human transmission rate from unit 4	.31(1.46)	Estimated
β_5	Human to human transmission rate from unit 5	.33(3.20)	Estimated
β'_2	Environment to human transmission rate from unit 2	.99(.98)	Estimated
β'_3	Environment to human transmission rate from unit 3	.80(10.40)	Estimated
β'_4	Environment to human transmission rate from unit 4	.60(6.72)	Estimated
β'_5	Environment to human transmission rate from unit 5	.07(3.64)	Estimated
σ	Rate of becoming infectious	1/5.2	(Assiri et al.,2013a)
γ	Recovery rate (not in the model)	1/25 –1/18	(Assiri et al.,2013a)
ρ	Rate of shedding virus into the environment	.27(.30)	Estimated
ϵ	Modulation of HCW for shedding into the environment	1(.11)	Estimated
ξ	Modulation of mobile HCW for shedding into the environment	.99(1.59)	Estimated
δ	Natural removal rate	1/2	(Assiri et al.,2013a)
c	Reduction factor for exposed	$.3(4 \times 10^{-4})$	Estimated

HCW (disease) Parameters

Table S3. HCW (disease) Parameters, Description and Base Values.

Parameter	Description	Base values (SD)	Sources
$\beta_{2,H}$	Human to Human transmission rate from unit 2 to a HCW	.49(.04)	Estimated
$\beta_{3,H}$	Human to Human transmission rate from unit 3 to a HCW	.01(.28)	Estimated
$\beta_{4,H}$	Human to Human transmission rate from unit 4 to a HCW	–	Excluded
$\beta_{5,H}$	Human to Human transmission rate from unit 5 to a HCW	.04(32)	Estimated
β_m	Human to Human transmission rate to a mobile HCW	$1 \times 10^{-3}(5 \times 10^{-3})$	Estimated
$\beta'_{2,H}$	Environment to human transmission rate from unit 2 to a HCW	.01(.07)	Estimated
$\beta'_{3,H}$	Environment to human transmission rate from unit 3 to a HCW	$3 \times 10^{-3}(.08)$	Estimated
$\beta'_{4,H}$	Environment to human transmission rate from unit 4 to a HCW	–	Excluded
$\beta'_{5,H}$	Environment to human transmission rate from unit 5 to a HCW	.64(1.33)	Estimated
β'_m	Environment to human transmission rate to a mobile HCW	$1 \times 10^{-3}(2 \times 10^{-3})$	Estimated

Next Generation Matrix to find R_0

We formulated the matrix $\mathcal{F}$ that consider the inputs due to infections and the matrix $\mathcal{V}$ that consider the outputs or movements of individuals. Then we found the two matrices at the DFE.

Matrix F

The infection matrix

$$\frac{\partial \mathcal{F}}{\partial E_2, ..., HE_m} = \begin{bmatrix} \mathcal{F}_{2,2} & \mathbf{0} & \mathbf{0} & \mathbf{0} & \mathcal{F}_{2,6} \\ \hline \mathbf{0} & \mathcal{F}_{3,3} & \mathbf{0} & \mathbf{0} & \mathcal{F}_{3,6} \\ \hline \mathbf{0} & \mathbf{0} & \mathcal{F}_{4,4} & \mathbf{0} & \mathcal{F}_{4,6} \\ \hline \mathbf{0} & \mathbf{0} & \mathbf{0} & \mathcal{F}_{5,5} & \mathcal{F}_{5,6} \\ \hline \mathcal{F}_{6,2} & \mathcal{F}_{6,3} & \mathcal{F}_{6,4} & \mathcal{F}_{6,5} & \mathcal{F}_{6,6} \end{bmatrix}$$

Where the block matrices are

$$\mathcal{F}_{2,2} = \begin{bmatrix} \beta_2 c - 2\beta_2 cE_2 - \beta_2(1+c)I_2 - \beta_2 I_2 - \beta_2 c\epsilon HE_2 - \beta_2 c\xi HE_m - \beta_2' V_2 & \beta_2 - \beta_2 cE_2 - \beta_2 E_2 - 2\beta_2 I_2 - \beta_2 c\epsilon HE_2 - \beta_2 c\xi HE_m - \beta_2' V_2 & \beta_2 c\epsilon - \beta_2 c\epsilon E_2 - \beta_2 c\epsilon I_2 & \beta_2' - \beta_2' E_2 - \beta_2' I_2 \\ 0 & 0 & 0 & 0 \\ \beta_{2,H} c - \beta_{2,H} cHE_2 & \beta_{2,H} - \beta_{2,H} HE_2 & \beta_{2,H} c\epsilon - \beta_{2,H} cE_2 - \beta_{2,H} I_2 - 2\beta_{2,H} c\epsilon HE_2 - \beta_{2,H} c\xi HE_m - \beta_{2,H}' V_2 & \beta_{2,H}' - \beta_{2,H}' HE_2 \\ 0 & 0 & 0 & 0 \end{bmatrix}$$

$$\mathcal{F}_{3,3} = \begin{bmatrix} \beta_3 c - 2\beta_3 cE_3 - \beta_3(1+c)I_3 - \beta_3 I_3 - \beta_3 c\epsilon HE_3 - \beta_3 c\xi HE_m - \beta_3' V_3 & \beta_3 - \beta_3 cE_3 - \beta_3 E_3 - 2\beta_3 I_3 - \beta_3 c\epsilon HE_3 - \beta_3 c\xi HE_m - \beta_3' V_3 & \beta_3 c\epsilon - \beta_3 c\epsilon E_3 - \beta_3 c\epsilon I_3 & \beta_3' - \beta_3' E_3 - \beta_3' I_3 \\ 0 & 0 & 0 & 0 \\ \beta_{3,H} c - \beta_{3,H} cHE_3 & \beta_{3,H} - \beta_{3,H} HE_3 & \beta_{3,H} c\epsilon - \beta_{3,H} cE_3 - \beta_{3,H} I_3 - 2\beta_{3,H} c\epsilon HE_3 - \beta_{3,H} c\xi HE_m - \beta_{3,H}' V_3 & \beta_{3,H}' - \beta_{3,H}' HE_3 \\ 0 & 0 & 0 & 0 \end{bmatrix}$$

$$\mathcal{F}_{4,4} = \begin{bmatrix} \beta_4 c - 2\beta_4 cE_4 - \beta_4(1+c)I_4 - \beta_4 I_4 - \beta_4 c\epsilon HE_4 - \beta_4 c\xi HE_m - \beta_4' V_4 & \beta_4 - \beta_4 cE_4 - \beta_4 E_4 - 2\beta_4 I_4 - \beta_4 c\epsilon HE_4 - \beta_4 c\xi HE_m - \beta_4' V_4 & \beta_4 c\epsilon - \beta_4 c\epsilon E_4 - \beta_4 c\epsilon I_4 & \beta_4' - \beta_4' E_4 - \beta_4' I_4 \\ 0 & 0 & 0 & 0 \\ \beta_{4,H} c - \beta_{4,H} cHE_4 & \beta_{4,H} - \beta_{4,H} HE_4 & \beta_{4,H} c\epsilon - \beta_{4,H} cE_4 - \beta_{4,H} I_4 - 2\beta_{4,H} c\epsilon HE_4 - \beta_{4,H} c\xi HE_m - \beta_{4,H}' V_4 & \beta_{4,H}' - \beta_{4,H}' HE_4 \\ 0 & 0 & 0 & 0 \end{bmatrix}$$

$$\mathcal{F}_{5,5} = \begin{bmatrix} \beta_5 c - 2\beta_5 cE_5 - \beta_5(1+c)I_5 - \beta_5 I_5 - \beta_5 c\epsilon HE_5 - \beta_5 c\xi HE_m - \beta_5' V_5 & \beta_5 - \beta_5 cE_5 - \beta_5 E_5 - 2\beta_5 I_5 - \beta_5 c\epsilon HE_5 - \beta_5 c\xi HE_m - \beta_5' V_5 & \beta_5 c\epsilon - \beta_5 c\epsilon E_5 - \beta_5 c\epsilon I_5 & \beta_5' - \beta_5' E_5 - \beta_5' I_5 \\ 0 & 0 & 0 & 0 \\ \beta_{5,H} c - \beta_{5,H} cHE_5 & \beta_{5,H} - \beta_{5,H} HE_5 & \beta_{5,H} c\epsilon - \beta_{5,H} cE_5 - \beta_{5,H} I_5 - 2\beta_{5,H} c\epsilon HE_5 - \beta_{5,H} c\xi HE_m - \beta_{5,H}' V_5 & \beta_{5,H}' - \beta_{5,H}' HE_5 \\ 0 & 0 & 0 & 0 \end{bmatrix}$$

$$\mathcal{F}_{6,6} = \begin{bmatrix} \beta_m c\xi - \beta_m cE_2 - \beta_m cE_3 - \beta_m cE_4 - \beta_m cE_5 - \beta_m c\epsilon HE_2 - \beta_m c\epsilon HE_3 - \beta_m c\epsilon HE_4 - \beta_m c\epsilon HE_5 - 2\beta_m c\xi HE_m - \beta_m I_2 - \beta_m I_3 - \beta_m I_4 - \beta_m I_5 - \beta_m' V_2 - \beta_m' V_3 - \beta_m' V_4 - \beta_m' V_5 \end{bmatrix}$$

$$\mathcal{F}_{2,6} = \begin{bmatrix} \beta_2 c\xi - \beta_2 E_2 c\xi - \beta_2 I_2 c\xi \\ 0 \\ \beta_{2,H} c\xi - \beta_{2,H} H E_2 c\xi \\ 0 \end{bmatrix}$$

$$\mathcal{F}_{3,6} = \begin{bmatrix} \beta_3 c\xi - \beta_3 E_3 c\xi - \beta_3 I_3 c\xi \\ 0 \\ \beta_{3,H} c\xi - \beta_{3,H} H E_3 c\xi \\ 0 \end{bmatrix}$$

$$\mathcal{F}_{4,6} = \begin{bmatrix} \beta_4 c\xi - \beta_4 E_4 c\xi - \beta_4 I_4 c\xi \\ 0 \\ \beta_{4,H} c\xi - \beta_{4,H} H E_4 c\xi \\ 0 \end{bmatrix}$$

$$\mathcal{F}_{5,6} = \begin{bmatrix} \beta_5 c\xi - \beta_5 E_5 c\xi - \beta_5 I_5 c\xi \\ 0 \\ \beta_{5,H} c\xi - \beta_{5,H} H E_5 c\xi \\ 0 \end{bmatrix}$$

$$\mathcal{F}_{6,2} = \begin{bmatrix} \beta_m c - \beta_m c H E_m & \beta_m - \beta_m H E_m & \beta_m c\epsilon - \beta_m c\epsilon H E_m & \beta'_m - \beta'_m H E_m \end{bmatrix}$$

$$\mathcal{F}_{6,3} = \begin{bmatrix} \beta_m c - \beta_m c H E_m & \beta_m - \beta_m H E_m & \beta_m c\epsilon - \beta_m c\epsilon H E_m & \beta'_m - \beta'_m H E_m \end{bmatrix}$$

$$\mathcal{F}_{6,4} = \begin{bmatrix} \beta_m c - \beta_m c H E_m & \beta_m - \beta_m H E_m & \beta_m c\epsilon - \beta_m c\epsilon H E_m & \beta'_m - \beta'_m H E_m \end{bmatrix}$$

$$\mathcal{F}_{6,5} = \begin{bmatrix} \beta_m c - \beta_m c H E_m & \beta_m - \beta_m H E_m & \beta_m c\epsilon - \beta_m c\epsilon H E_m & \beta'_m - \beta'_m H E_m \end{bmatrix}$$

At the DFE $\mathcal{F}'$ is the matrix F given by

$$F = \mathcal{F}'|_{DFE} = \begin{bmatrix} F_{2,2} & \mathbf{0} & \mathbf{0} & \mathbf{0} & F_{2,6} \\ \mathbf{0} & F_{3,3} & \mathbf{0} & \mathbf{0} & F_{3,6} \\ \mathbf{0} & \mathbf{0} & F_{4,4} & \mathbf{0} & F_{4,6} \\ \mathbf{0} & \mathbf{0} & \mathbf{0} & F_{5,5} & F_{5,6} \\ F_{6,2} & F_{6,3} & F_{6,4} & F_{6,5} & F_{6,6} \end{bmatrix}$$

where

$$F_{i,i} = \begin{bmatrix} \beta_i c & \beta_i & \beta_i c\epsilon & \beta_i' \\ 0 & 0 & 0 & 0 \\ \beta_{i,H} c & \beta_{i,H} & \beta_{i,H} c\epsilon & \beta_{i,H}' \\ 0 & 0 & 0 & 0 \end{bmatrix}$$

for $i = 2, 3, 4, 5$, and

$$F_{6,6} = \begin{bmatrix} \beta_m c\xi \end{bmatrix}.$$

Whereas

$$F_{i,6} = \begin{bmatrix} \beta_i c\xi \\ 0 \\ \beta_{i,H} c\xi \\ 0 \end{bmatrix}$$

and

$$F_{6,i} = \begin{bmatrix} \beta_m c & \beta_m & \beta_m c\epsilon & \beta_m' \end{bmatrix}$$

for $i = 2, 3, 4, 5$.

Matrix V

The matrix

$$\mathcal{V} = \begin{bmatrix} -(\alpha_{1,2}E_1 + \alpha_{3,2}E_3 + \alpha_{4,2}E_4 + \alpha_{5,2}E_5) + (\alpha_{2,1} + \alpha_{2,3} + \alpha_{2,4} + \alpha_{2,5})E_2 + \sigma E_2 \\ -(\sigma E_2 + \alpha_{1,2}I_1 + \alpha_{3,2}I_3 + \alpha_{4,2}I_4 + \alpha_{5,2}I_5) + (\alpha_{2,3} + \alpha_{2,4} + \alpha_{2,5} + \alpha_{2,6})I_2 \\ \sigma H E_2 \\ -\rho(c(E_2 + \epsilon H E_2 + \xi H E_m) + I_2) + (b_2 + \delta)V_2 \\ \hline -(\alpha_{1,3}E_1 + \alpha_{2,3}E_2 + \alpha_{4,3}E_4 + \alpha_{5,3}E_5) + (\alpha_{3,1} + \alpha_{3,2} + \alpha_{3,4} + \alpha_{3,5})E_3 + \sigma E_3 \\ -(\sigma E_3 + \alpha_{1,3}I_1 + \alpha_{2,3}I_2 + \alpha_{4,3}I_4 + \alpha_{5,3}I_5) + (\alpha_{3,2} + \alpha_{3,4} + \alpha_{3,5} + \alpha_{3,6})I_3 \\ \sigma H E_3 \\ -\rho(c(E_3 + \epsilon H E_3 + \xi H E_m) + I_3) + (b_3 + \delta)V_3 \\ \hline -(\alpha_{1,4}E_1 + \alpha_{2,4}E_2 + \alpha_{3,4}E_3 + \alpha_{5,4}E_5) + (\alpha_{4,1} + \alpha_{4,2} + \alpha_{4,3} + \alpha_{4,5})E_4 + \sigma E_4 \\ -(\sigma E_4 + \alpha_{1,4}I_1 + \alpha_{2,4}I_2 + \alpha_{3,4}I_3 + \alpha_{5,4}I_5) + (\alpha_{4,2} + \alpha_{4,3} + \alpha_{4,5} + \alpha_{4,6})I_4 \\ \sigma H E_4 \\ -\rho(c(E_4 + \epsilon H E_4 + \xi H E_m) + I_4) + (b_4 + \delta)V_4 \\ \hline -(\alpha_{1,5}E_1 + \alpha_{2,5}E_2 + \alpha_{3,5}E_3 + \alpha_{4,5}E_4) + (\alpha_{5,1} + \alpha_{5,2} + \alpha_{5,3} + \alpha_{5,4})E_5 + \sigma E_5 \\ -(\sigma E_5 + \alpha_{1,5}I_1 + \alpha_{2,5}I_2 + \alpha_{3,5}I_3 + \alpha_{4,5}I_4) + (\alpha_{5,2} + \alpha_{5,3} + \alpha_{5,4} + \alpha_{5,6})I_5 \\ \sigma H E_5 \\ -\rho(c(E_5 + \epsilon H E_5 + \xi H E_m) + I_5) + (b_5 + \delta)V_5 \\ \hline \sigma H E_m \end{bmatrix}$$

Matrix for V which is equal to $\mathcal{V}'$ at the DFE

$$V = \mathcal{V}' = \begin{bmatrix} V_{2,2} & V_{2,3} & V_{2,4} & V_{2,5} & V_{2,6} \\ V_{3,2} & V_{3,3} & V_{3,4} & V_{3,5} & V_{3,6} \\ V_{4,2} & V_{4,3} & V_{4,4} & V_{4,5} & V_{4,6} \\ V_{5,2} & V_{5,3} & V_{5,4} & V_{5,5} & V_{5,6} \\ V_{6,2} & V_{6,3} & V_{6,4} & V_{6,5} & V_{6,6} \end{bmatrix} \tag{1}$$

Where the block matrices are

where

$$V_{i,i} = \begin{bmatrix} \sum_{j \neq i} \alpha_{i,j} + \sigma & 0 & 0 & 0 \\ -\sigma & \sum_{j \neq 1,i} \alpha_{i,j} & 0 & 0 \\ 0 & 0 & \sigma & 0 \\ -c & -1 & -c\epsilon & b_i + \delta \end{bmatrix}$$

for $i = 2, 3, 4, 5$

$$V_{i,j} = \begin{bmatrix} -\alpha_{j,i} & 0 & 0 & 0 \\ 0 & -\alpha_{j,i} & 0 & 0 \\ 0 & 0 & 0 & 0 \\ 0 & 0 & 0 & 0 \end{bmatrix}$$

for $i, j = 2, 3, 4, 5$ and $i \neq j$. Whereas

$$V_{i,6} = \begin{bmatrix} 0 \\ 0 \\ 0 \\ -c\xi \end{bmatrix}$$

and

$$V_{6,i} = \begin{bmatrix} 0 & 0 & 0 & 0 \end{bmatrix}$$

for $i = 2, 3, 4, 5$, and

$$V_{6,6} = \begin{bmatrix} \sigma \end{bmatrix}$$

References

1. Nowotny N, Kolodziejek J. Middle East respiratory syndrome coronavirus (MERS-CoV) in dromedary camels, Oman, 2013. Euro Surveill. 2014;19(16):20781.
2. Reusken CBEM, Haagmans BL, Müller MA, Gutierrez C, Godeke G-J, Meyer B, et al. Middle East respiratory syndrome coronavirus neutralising serum antibodies in dromedary camels: a comparative serological study. The Lancet Infectious Diseases. 2013;13(10):859-66.
3. Alshukairi AN, Zheng J, Zhao J, Nehdi A, Baharoon SA, Layqah L, et al. High Prevalence of MERS-CoV Infection in Camel Workers in Saudi Arabia. MBio. 2018;9(5).
4. Al-Dorzi HM, Aldawood AS, Khan R, Baharoon S, Alchin JD, Matroud AA, et al. The critical care response to a hospital outbreak of Middle East respiratory syndrome coronavirus (MERS-CoV) infection: an observational study. Ann Intensive Care. 2016;6(1):101.
5. Mackay IM, Arden KE. MERS coronavirus: diagnostics, epidemiology and transmission. Virol J. 2015;12:222.
6. Kasem S, Qasim I, Al-Doweriej A, Hashim O, Alkarar A, Abu-Obeida A, et al. The prevalence of Middle East respiratory Syndrome coronavirus (MERS-CoV) infection in livestock and temporal relation to locations and seasons. J Infect Public Health. 2018;11(6):884-88.
